# 3-D Culture of Marine Sponge Cells for Production of Bioactive Compounds

**DOI:** 10.3390/md19100569

**Published:** 2021-10-14

**Authors:** Elizabeth Urban-Gedamke, Megan Conkling, Peter J. McCarthy, Paul S. Wills, Shirley A. Pomponi

**Affiliations:** Harbor Branch Oceanographic Institute, Florida Atlantic University, 5600 US Highway 1 North, Fort Pierce, FL 34946, USA; urbane@fau.edu (E.U.-G.); mconkli2@fau.edu (M.C.); pmccart5@fau.edu (P.J.M.); pwills2@fau.edu (P.S.W.)

**Keywords:** marine sponge, 3-D culture, FibraCel^®^ disks, hydrogel, ultra low temperature agarose, gel microdroplets, marine natural products

## Abstract

Production of sponge-derived bioactive compounds in vitro has been proposed as an alternative to wild harvest, aquaculture, and chemical synthesis to meet the demands of clinical drug development and manufacture. Until recently, this was not possible because there were no marine invertebrate cell lines. Recent breakthroughs in the development of sponge cell lines and rapid cell division in improved nutrient media now make this approach a viable option. We hypothesized that three-dimensional (3-D) cell cultures would better represent how sponges function in nature, including the production of bioactive compounds. We successfully cultured sponge cells in 3-D matrices using FibraCel^®^ disks, thin hydrogel layers, and gel microdroplets (GMDs). For in vitro production of bioactive compounds, the use of GMDs is recommended. Nutrients and sponge products rapidly diffuse into and out of the 3-D matrix, the GMDs may be scaled up in spinner flasks, and cells and/or secreted products can be easily recovered. Research on scale-up and production is in progress in our laboratory.

## 1. Introduction

Sponges are one of the most prolific sources of marine natural products (MNPs) [1]. Unfortunately, many pharmaceutically relevant MNPs are found only in trace amounts within the source sponge [2,3], and it is neither economically nor ecologically feasible to harvest enough wild sponge biomass to supply the necessary quantities for clinical drug development and manufacture [3]. In situ aquaculture of whole sponges or sponge “explants” (fragments) has been successful in situ for a limited number of species [4], however, the inability to control environmental conditions (e.g., extreme weather events, harmful algal blooms, etc.) makes in situ aquaculture a less desirable biological option.

In vitro cultivation of sponge cells is another biological option for production of biomass or bioactive metabolites [5]. Due to their cellular organization, sponges can be dissociated into cells that will reaggregate and differentiate to form a functional sponge [6]. Cell culture allows for precise control of environmental variables and selection or optimization of conditions that favor increased production of biomass and/or bioactive metabolites. Typically, normal (mammalian) cells form a monolayer and remain attached to the substrate to proliferate. Increased understanding of basic metabolic processes at the cellular level in mammalian cell cultures has led to a transition to understanding these processes in differentiated, three-dimensional (3-D) populations of cells [7]. Cells in two-dimensional (2-D) culture exhibit different morphological and physiological characteristics, such as changes in functionality, morphology, phenotype, and metabolic activity [7]. Cell-to-cell and cell-to-extracellular matrix (ECM) interactions play a key role in these characteristics and are limited in 2-D culture [7].

While there is still much to learn about basic cellular and molecular processes in sponge cells, there is much to gain from research using sponge cell and “tissue” cultures, e.g., primmorphs [8]. The aim of this study was to evaluate sponge cells cultured in three 3-D substrates: FibraCel^®^ disks, thin hydrogel layers, and gel microdroplets (GMDs), with the goal of applying one or more of these methods to scale-up production of sponge biomass and/or bioactive metabolites.

FibraCel^®^ disks (Eppendorf, Enfield, CT, USA) are solid support matrices made of a nonwoven polyester mesh and a polypropylene support grid that are treated to attract cells to attach to the disk [9]. The porous nature of the nonwoven mesh increases the surface area available for cell attachment because the cells can infiltrate the mesh and attach on the inner surfaces as well as the outside of the disk [10]. FibraCel^®^ disks are designed for use in cell culture bioreactors, which allows for long-term high density cell cultures in perfusion mode. This is particularly advantageous for the production of biologically derived compounds. We have previously demonstrated that cells of the sponge *Axinella corrugata* could be immobilized on FibraCel^®^ disks, and that the cultured cells continued to produce stevensine [11].

Hydrogels, like gelatin and agarose, are crosslinked polymer networks with high water content [12]. To form a hydrogel, a liquid precursor solution seeded with living cells is polymerized using physical or chemical crosslinking [12]. The gels imitate elements of native ECMs, including exhibiting mechanics similar to soft tissues and promoting cellular adhesion. These properties increase the appeal of hydrogels as a surrogate ECM [12].

The properties of hydrogels are translatable to GMDs, which are created using the same materials (gelatin, agarose). GMDs are small spheres of hydrogel in which few or even single cells can be encapsulated [12]. The small volume of GMDs makes them highly permeable, which allows for communication among cells and the diffusion of cellular metabolites [12,13]. Culturing marine sponge cells presents some unique challenges, for example, marine sponges require high salinities which can prevent the hydrogels from solidifying properly. In addition, many hydrogels require curing periods at high temperatures that are lethal to sponge cells.

Researchers have made extensive efforts to create a sponge cell line [3], but until recently only primary cell cultures with a finite lifespan have been established. With the development of an improved nutrient medium [14], Conkling et al. [15] demonstrated exceptionally rapid rates of cell division in several species of sponges and developed finite cell lines for three species of the genus *Geodia*. These recent advancements had not yet been applied to 3-D sponge cell culture. We hypothesized that combining these methods, i.e., improved nutrient media and the use of 3-D matrices, to obtain rapidly dividing cells in a 3-D system would increase cell-to-cell and cell-to-ECM communication and would be more similar to how sponges function in nature, including the production of bioactive compounds.

## 2. Results

### 2.1. 2-D Cultures of Geodia neptuni Were Used as Controls

**Figure 1:**
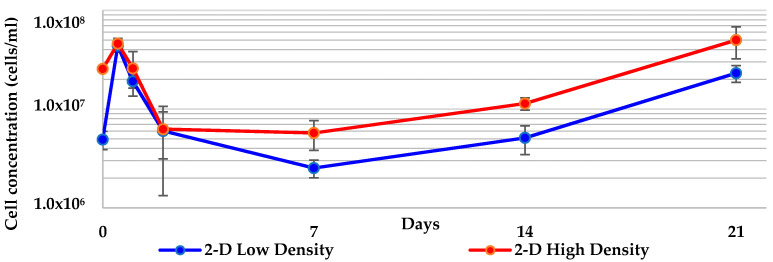
Cell concentrations of the two inoculation densities in 2-D control cultures. Cell numbers increased from two to nearly five times the initial concentration over the 21-day culture period.

To compare cell division in the 3-D treatments with what has been reported previously in 2-D cultures, *Geodia neptuni* cells were cultured at the two densities used for all 3-D treatments: low density (5 × 10^6^ cells/mL) and high density (1 × 10^7^ cells/mL). Cell concentrations increased over the first 12 h in the 2-D control cultures, as previously reported [15] (Figure 1). The extent of that increase was inversely related to the initial inoculation density—cells at the lower inoculation density increased the most dramatically while cells at the higher inoculation density increased the least. This finding supports the hypothesis that the high density treatments are nutrient limited. The carrying capacity of the medium provides lower density treatments with more nutrients per unit volume than the higher density treatments. The same is true for the decrease seen in both treatments between 12 and 48 h. By day 7 both treatments began a second, slow increase which continued through the end of the experimental culture period.

While both treatments exhibited higher densities on day 21 than the inoculation density, the low density treatment reached its highest density at 12 h (Figure 1), nearly an order of magnitude increase in cell number. There was no significant difference between treatments when the final or maximum densities of each treatment were compared (Table 1).

A monolayer formed on the surface of each well. Over time, the cultures darkened due to the increased number of cells present and to the pigmentation cells acquired during cell division in M1 medium [15]. The cells also formed aggregates. No evidence of cell differentiation or the formation of adult sponge architecture was seen. Nevertheless, the increase in cell number ranged from two to nearly five times the initial concentration over the 21-day culture period (Figure 1).

### 2.2. Geodia neptuni Cells Can Be Cultured in FibraCel^®^ Disks

After 12 h of incubation in M1 medium, *Geodia neptuni* cells attached to the polyester mesh of the FibraCel^®^ disk. The density of cells visible on the polyester fibers increased over time, however, it was difficult to get accurate cell counts. Cells could not be completely removed from the FibraCel^®^ disks using calcium- and magnesium-free seawater (CMF) and agitation.

**Figure 2:**
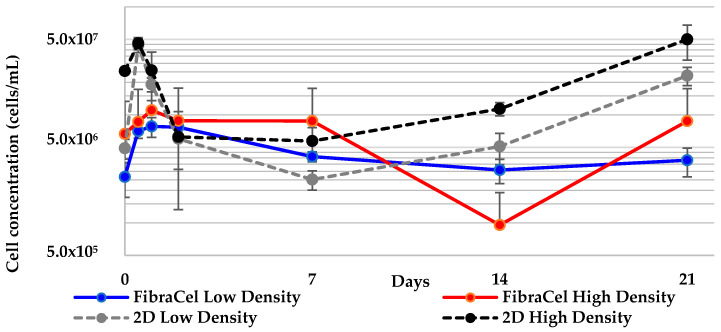
Cell concentrations for the two inoculation densities of cells cultured in FibraCel^®^ disks over time, compared to the 2-D controls. The total number of cells at each time point are underrepresented, due to the inability to completely remove all cells from the disks.

Visual (microscopic) inspection of the disks before and after soaking in CMF to remove the cells showed little difference in the quantity of cells attached to the mesh fibers. Large aggregates and distinctive clusters of cells were microscopically imaged before soaking in CMF and those same cells were observed attached in the same place after soaking in CMF (images not shown). This result demonstrates that soaking in CMF is not a reliable method to remove all attached sponge cells from FibraCel^®^ disks. Cell counts indicate that some of the cells can be removed using CMF, but the number was much lower than expected; many cells remained attached to the mesh, rendering the counts inaccurate (Figure 2).

**Figure 3:**
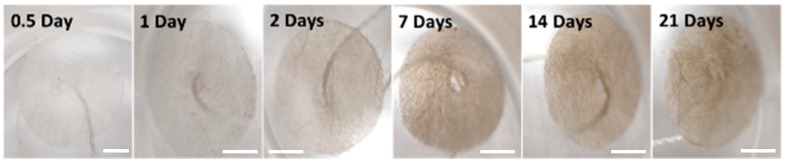
Pigmented dividing cells attached to the polyester fibers cause the FibraCel^®^ disks to also appear pigmented. Increasing pigmentation of the FibraCel^®^ disks over time indicates an increase in cell number. Scale bars = 10 mm.

As previously reported [15], *G. neptuni* cells become pigmented when cultured in medium M1. The increased number of cells attached to the polyester disk and the dark pigmentation of those cells causes the disk itself to appear pigmented (Figure 3). After the cells were removed by soaking in CMF, the cells still attached to the disks remained pigmented. This, along with the microscopic evaluation of the disks showing cells still attached to the mesh, was taken as an indication that a large number of cells remained attached to the polyester mesh. It was also observed that the disks darkened over the 21-day culture period (Figure 3).

**Figure 4:**
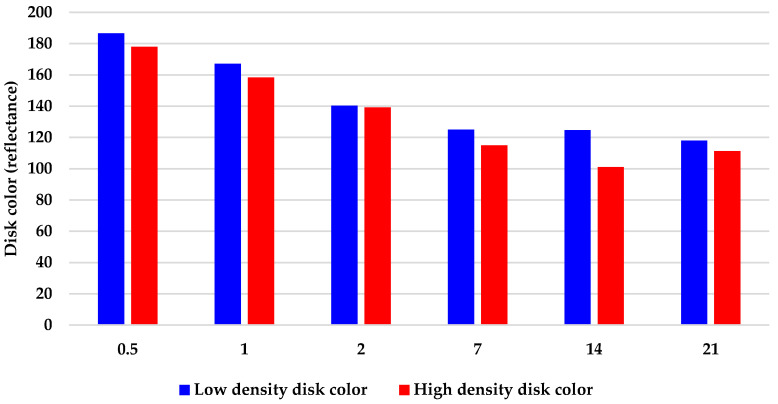
Changes in pigmentation of FibraCel^®^ disks after partial cell removal by soaking in calcium- and magnesium-free seawater (CMF), separated into color codes on a scale of 0–200 (200 = white, 0 = black). Decreasing values indicate darker disk color (and more cells attached to disks).

As an alternative to cell counts (which were not accurate) and total protein analyses (which could not be measured because of interference of the pigment with the protein assay) (Appendix A), the disks were photographed after cell removal and counting, and the resulting images were analyzed to quantify the change in pigment over time. The average pixel coloration value decreased over time in culture, corresponding to the darkening of the disks (Figure 4).

While the cell counts were deemed unreliable, the maximum and final cell concentrations were compared between treatment groups and between the corresponding 2-D control culture inoculation densities. The differences between low density FibraCel^®^ treatments at final density and maximum density were found to be significantly different from the 2-D low density treatment (Table 2). Despite the potential for underestimation of cell counts, cell numbers in both high and low density treatments increased, as indicated by the pigmented FibraCel^®^ disks (Figure 3).

### 2.3. Porcine Gelatin Layers Dissolve in M1 Medium

Cells were immobilized in microbial transglutaminase (mTGase) crosslinked porcine gelatin and cultured for 21 days. Cell counts in the high cell density treatment remained higher than those in the low cell density treatment for the duration of the culture period (data not shown). However, cell counts only increased within the first week of the experiment. The low cell density treatment showed a decrease in cell number between days 2 and 7, while the cell number in the high cell density treatment decreased between days 7 and 14. This decrease in average cell number corresponded with images taken during the experiment that showed that the gels began to dissolve as early as day 7 (data not shown). Following the dissolution of the porcine gelatin hydrogels during the 21-day culture experiment, two attempts at optimization were made—increasing the crosslinking incubation time to increase the stability of the gel and increasing the volume of the gel to decrease the surface to volume ratio. Neither of these resulted in hydrogels that lasted the full 21-day period. Due to this, ultra low temperature agarose (ULTA) was used in subsequent experiments.

### 2.4. Geodia neptuni Cells Can Be Cultured in ULTA Thin Hydrogel Layers

**Figure 5:**
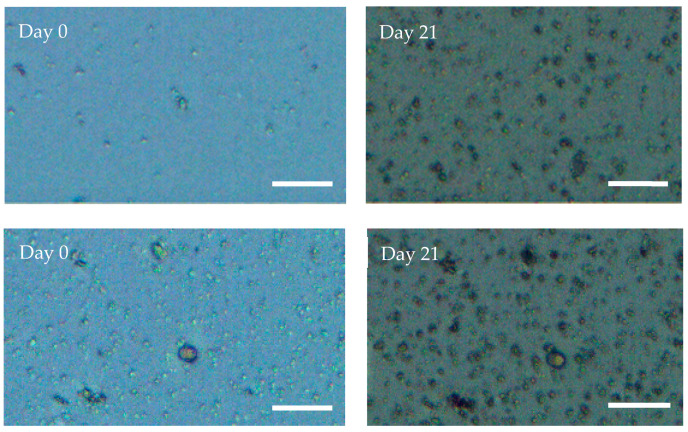
Z-stack images show an increase in individual and aggregated *Geodia neptuni* cells cultured in ultra low temperature agarose (ULTA). Despite the small size of *Geodia neptuni* cells (≤2 μm), an increase in both cell density and pigmentation is apparent for both densities between day 0 and day 21 in culture. Top row: low density treatment, bottom row, high density treatment. Scale bar = 25 µm.

**Figure 6:**
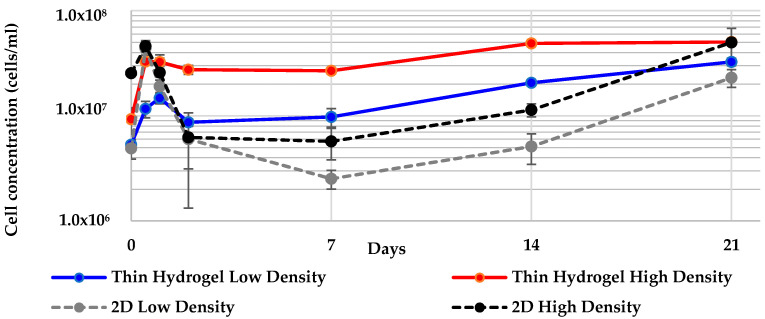
Cell concentration over 21-day culture of *G. neptuni* cells in ULTA thin layers, compared to 2-D controls. Both treatments showed an overall increase in final cell counts when compared to the inoculation density.

Cells were successfully cultured in thin layers of ULTA. Z-stack micrographs show a marked increase in cells from day 0 to day 21 in both cell densities (Figure 5). As with the FibraCel^®^ disk cultures, the ULTA thin layer cultures also exhibited an increase in pigmentation over time. Cells recovered from the gels showed an increase in cell number in both treatments in the first 12 h (Figure 6). This increase continued for the low density treatment until 24 h, while cell number in the high density treatment decreased slightly. Both treatments recovered and began increasing again slowly after 7 days (Figure 6). Cell counts of the spent medium showed that at no point in the experiment did the number of cells removed with the medium exceed 2% of the cells counted within the gel. Both treatments showed an overall increase in final cell counts when compared to the inoculation density (Figure 6).

The maximum cell concentration for both the low and high density ULTA layer cultures was reached at day 21. The only significant difference detected was for the final cell concentration between the high and low density thin hydrogel treatments (Table 3.)

### 2.5. Geodia neptuni Cells Can Be Cultured in ULTA GMDs

**Figure 7:**
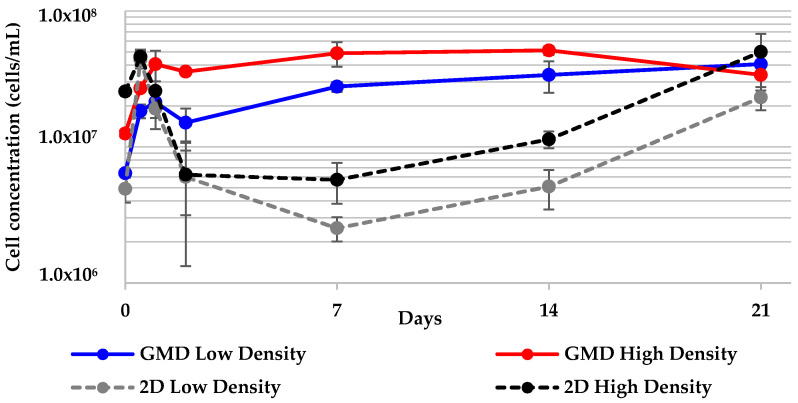
Concentration of *G. neptuni* cells per mL of ULTA cultured for 21 days in gel microdroplets (GMDs), compared to 2-D controls. All cell density treatments reached a plateau after 24 h and all reached a similar cell concentration by the end of 21 days.

Cells were successfully cultured in 10 µL ULTA GMDs for 21 days. Throughout the culture period, cell number increased most dramatically over the first 12 h in all treatments (Figure 7). All cell density treatments reached a plateau after 24 h and all reached a similar cell concentration by the end of 21 days. The low density treatment reached a maximum cell concentration on day 21, while the high density treatment reached its maximum cell density on day 14.

**Figure 8:**
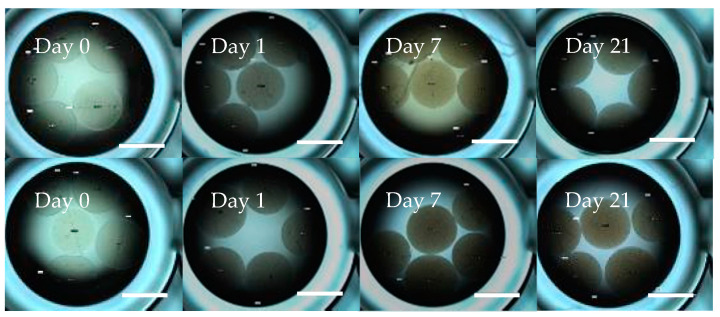
Darkening of ULTA GMDs containing *G. neptuni* cells indicates cell division due to increasing pigmentation of cells as they divide. GMDs are cultured in M1 medium in a 96-well plate. Top row: low density treatment; bottom row: high density treatment. Scale bar = 2 mm.

All GMD treatments were compared between themselves as well as to the corresponding 2-D control treatments. No statistically significant difference was found in any of the pairings (Table 4). The pigment of the GMD cultures became darker over time (Figure 8). No evidence of droplet dissolution was observed throughout this experiment. The average droplet diameter for all treatment groups changed by <4.0% (data not shown).

### 2.6. All 3-D Treatments Resulted in Substantial Increases in Cell Numbers

**Figure 9:**
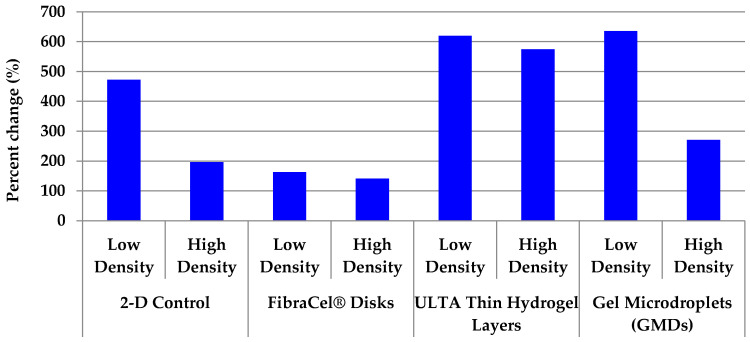
Comparison of increases in cell number for all three-dimensional (3-D) treatments over 21 days. Cell numbers increased in all treatments throughout the culture period. The greatest increase was observed in the low density treatments. Due to the inability to remove all cells from the disks, the increase in cell numbers in the FibraCel^®^ cultures is underrepresented in this graph.

Cells cultured in ULTA thin gel layers and GMDs exhibited as much as a 6-fold increase (low density treatments) in cell number over the course of the 21-day culture period (Figure 9). The low cell density treatment, regardless of culture method, exhibited higher percent change than the high cell density treatment. This suggests that nutrient limitation is a factor in these cultures. Percent increases for FibraCel^®^ disks are inaccurate (underrepresented) because of incomplete recovery of cells from the disks.

## 3. Discussion

Each 3-D method presents unique combinations of advantages and weaknesses (Table 5).

The major limitation of FibraCel^®^ disks is the inability to quantify cells. The most direct way to determine cell concentration is by counting cells recovered from the 3-D matrix. Complete removal of cells from FibraCel^®^ disks was not possible. The cells could not be removed from the mesh using CMF, which is a widely accepted method of disaggregating sponge cells. Cell recovery was not a problem with the other treatments because the ULTA hydrogel matrix itself could be digested enzymatically. Any agent capable of digesting the polyester FibraCel^®^ mesh would have also destroyed the cells. In addition, the fibers of the mesh obscured cells and aggregates within the disks, which made accurately counting cells difficult. As an alternative, total protein analysis was attempted as a proxy to determine an increase in cell numbers. As previously reported [15], the cells become pigmented when cultured in M1 medium, and the pigment interferes with protein measurements, which are based on light wavelength, rendering the total protein analysis assay useless for quantifying protein from these cells. An increase in overall pigmentation of the FibraCel^®^ disks using colorimeter software as a proxy for quantitative data suggested an increase in cell concentration, however, these data are qualitative. In conclusion, FibraCel^®^ disks are not recommended for any sponge cell culture applications using M1 medium that require precise counts of cell concentration. The hardiness of these disks and their ability to retain cells, however, make them a promising candidate for production of sponge biomass via aquaculture. FibraCel^®^ disks can be seeded with cells and could be transplanted to an aquaculture system for scale-up of biomass. FibraCel^®^ disks are also ideal candidates for production of bioactive compounds in vitro. They are designed for use in a packed-bed bioreactor, so a large quantity of cells can be cultured, and the desired natural products can be removed with the spent medium or harvested cell biomass [11]. This process is designed to be scaled-up. In addition, the process of seeding FibraCel^®^ disks with cells requires minimal effort and the disks themselves are commercially available and inexpensive.

Like FibraCel^®^ disks, cells cultured in ULTA thin hydrogel layers may be transplanted to a land-based aquaculture system for scale-up of biomass production. However, the ULTA hydrogel matrix is more delicate than FibraCel^®^ disks and would need to be placed in aquaculture tanks or raceways with gentle water movement. Although the ULTA thin hydrogel layers did not dissolve throughout the culture period, as was observed with the porcine gelatin layers, the matrix will degrade over time. This is a potentially limiting factor for aquaculture purposes, as the gel may not remain intact until the sponge cultures have stabilized and can be attached to a more robust substrate. The advantage of using ULTA thin hydrogel layers in vitro is the ability to monitor individual cells and aggregates that are immobilized in place over an extended period of time. Due to the clarity of the ULTA hydrogel, layers of cells can be microscopically imaged, and the individual layers can be stacked and analyzed three-dimensionally using software such as Image J. This feature can be especially useful for studying sponge cell differentiation and the formation of adult sponge architecture. The ULTA thin hydrogel layer method is more labor intensive than the FibraCel^®^ method: temperature and time requirements for forming the ULTA thin hydrogel layers must be balanced with the temperature limits of sponge cells, which lose viability when exposed to temperatures above 37 °C.

Many of the same characteristics of the ULTA thin hydrogel layers are translatable to GMDs, which are made of the same material (ULTA). As the ULTA GMDs are delicate and degrade over time, they are recommended for in vitro research. For example, sponge cells cultured in ULTA GMDs could be applied to in vitro production of MNPs, due to the rapid diffusion of medium and sponge products into and out of the matrix and to the ability to culture and scale-up the droplets in spinner flasks. ULTA GMDs would be useful for studying sponge cell metabolism for the same reasons. This method may also be used to create GMDs with single cells by serial dilution to study cell division, differentiation, and formation of 3-D architecture in the GMD small-volume microenvironment using high content imaging and/or flow cytometric analyses. The formation of ULTA GMDs is the most labor intensive of the methods evaluated. Therefore, scale-up may be challenging. Future studies using GMDs will require the development of an automated method to form the droplets, which will increase the consistency of droplet size and decrease the time and effort to create the GMDs.

Shared trends in cell concentration over time in all methods indicate nutrient limitation. The initial rapid increase of cells appears to exceed the carrying capacity of the medium, resulting in a decrease and then slow increase towards a plateau at a cell concentration that can be supported by the medium. This is further supported by the fact that all 3-D cultures reached similar final cell concentrations (3.26–8.55 × 10^7^). Further studies using perfusion culture, in which fresh medium is constantly added to the culture vessel while spent medium is removed, may enable a greater increase in cell numbers.

Before moving forward with further research using the original M1 or modified versions of M1 medium, it will be necessary to understand what is causing the pigmentation in cells cultured with this medium. In addition to interfering with light wavelength-based assays and measurements, the pigmentation also interferes with the ability to observe the cells or their components with fluorescent dyes. The inability to use these assays severely limits the ways in which marine sponge cells can be studied and the questions researchers can investigate. This pigmentation has been observed in a number of sponge species, including three species belonging to the genus *Geodia* [15]. Conkling et al. [15] hypothesized that this pigmentation is caused by the increased production of melanin, possibly due to some components of M1 medium.

It is important to note that M1 medium was first optimized for a different sponge species (*Dysidea etheria*) and only for short term (48 h) culture [14]. Additional optimization of M1 medium for long term culture may also be beneficial in inducing cell differentiation. An optimized version of the M1 medium (OpM1) [16] contains various growth factors, vitamins, and fetal bovine serum and has been shown to increase the maximum cell density and number of cell population doublings in 2-D cultures of the related species *Geodia barretti* [16]. Combining this medium with 3-D culture methods for *G. neptuni* has yet to be attempted and may produce favorable results.

## 4. Materials and Methods

### 4.1. Specimen Collection, Cell Dissociation and Cryopreservation

The marine sponge *Geodia neptuni* was selected for this research to demonstrate proof of concept of 3-D culture methods. Previous research has demonstrated that species of the genus *Geodia* showed less variation between individuals when compared to other sponge species, and of the *Geodia* species evaluated by Conkling et al. [15], *G. neptuni* exhibited minimal individual variation. *Geodia neptuni* also has been shown to divide rapidly in 2-D cell culture, making it a viable candidate for 3-D cell culture [15].

Three individuals of *Geodia neptuni* were sampled using scuba off Looe Key in the lower Florida Keys under Florida Keys National Marine Sanctuary (FKNMS) permit number FKNMS-2014-070 (to S.A.P.). The samples were kept in seawater during transport back to the shore-based laboratory facility on Summerland Key (Mote Marine Laboratory, Elizabeth Moore International Center for Coral Reef Research and Restoration) where they were dissociated and cryopreserved.

Cells from *G. neptuni* were dissociated and cryopreserved immediately after sampling using previously established methods [11,14,15]. The samples were cleaned of debris and associated macroorganisms before being cut into small (≤1 cm^3^) fragments with a scalpel. The fragments were transferred to sterile gauze and squeezed into a petri dish containing 30 mL filtered sea water (FSW) (0.2 µm filter) to release the cells. The cell suspension was filtered through a 70 µm cell strainer (Thermo Fisher Scientific, Waltham, MA, USA) to remove additional debris and large aggregates. The cells were centrifuged twice at 300× *g* for five minutes and resuspended in FSW each time.

Cell counts were taken using a Countess II FL Automated Cell Counter (Thermo Fisher Scientific, Waltham, MA, USA). Cell density was adjusted accordingly, the cell suspension was centrifuged at 300× *g* for five minutes, the supernatant was removed, the pellet was re-suspended in cryoprotectant (10% dimethyl sulfoxide (Sigma Aldrich, St. Louis, MO, USA) and 10% fetal bovine serum (R&D Systems, Minneapolis, MN, USA) in FSW), and 1 mL of the cell suspension was aliquoted into cryogenic vials and cooled to −80 °C at a rate of approximately −1 °C/min using Mr. Frosty^TM^ containers (Nalgene^®^, Rochester, NY, USA) [17].

### 4.2. Cell Preparation, Counting, and Imaging

To establish cell cultures, cryopreserved cells were rapidly thawed in a 50 °C water bath and cryoprotectant was removed by washing the cells twice in artificial sea water (ASW) and centrifuging (300× *g* for five minutes) to pellet the cells [17]. The rinsed cells were then suspended in ASW, and automated cell counts were made as described above. The cell concentration was adjusted to the desired density. Cell densities of 5 × 10^6^ and 1 × 10^7^ cells/mL were used. The lower density was selected based on previous studies by Conkling et al. [15], and the higher density was chosen to increase the possibility of cell-to-cell contact and facilitate cell aggregation. Microscopic images were taken at each timepoint using the EVOS Cell Imaging System (Thermo Fisher Scientific, Waltham, MA, USA) at a range of magnifications (20X–200X). For the ULTA gel layer and GMD methods, Z-stack micrographs were also taken. Evaluation of protein concentration using the BCA Protein Assay (Thermo Fisher Scientific, Waltham, MA, USA) was attempted as a proxy to cell counts (see Appendix A for details of methods and results).

### 4.3. Solution Preparation

#### 4.3.1. Media

Medium M1 (Marine 199 + salts, amino acids and antibiotics/antimycotics), optimized for the marine sponge *Dysidea etheria* [14], was used in all experimental procedures.

#### 4.3.2. Hydrogels

##### Porcine Gelatin

Artificial seawater (ASW) was heated to 37 °C and combined with porcine gelatin (Thermo Fisher Scientific, Waltham, MA, USA, 6.5% *w*/*v*) and 0.1% mTGase (Sigma Aldrich, St. Louis, MO, USA, 10% *v*/*v*). Thawed cryopreserved cells (as described above) were added to the gelatin at the two experimental cell densities (5 × 10^6^ and 1 × 10^7^ cells/mL). Gelatin was cooled to 35 °C before cells were added. Following the addition of cells, the gelatin was added to a 96-well plate (Falcon) and incubated at 4 °C for 30 min to solidify. A total of 100 µL of 0.3% *w*/*v* mTGase solution were added to each well, and the gelatin was incubated in this crosslinking solution for 30 min at room temperature. The mTGase was then removed via pipetting before the addition of nutrient medium.

##### ULTA

Artificial seawater (ASW) was heated to 70 °C and combined with ULTA (Sigma Aldrich, St. Louis, MO, USA, 2.5% *w*/*v*). The solution was cooled to 35 °C, and 1 mL of cells suspended in ASW was added and mixed thoroughly. The ULTA was cooled for 30 min at 4 °C to solidify.

#### 4.3.3. CMF

Salts were added to deionized water (449 mM sodium chloride, 9 mM potassium chloride, 33 mM sodium sulfate, 2.15 mM sodium bicarbonate, 10 mM Tris hydrochloride, 2.5 mM ethylenediaminetetraacetic acid). The resulting CMF solution was stirred on low heat until all added salts dissolved. The solution was cooled to room temperature, transferred to a sterile 1 L media vessel, and autoclaved. The sterile CMF was then stored at 4 °C.

### 4.4. Incubation of Cell Cultures

All cultures were incubated at room temperature, approximately 22 °C, and in the dark to protect the media from light [14,15]. Cells were cultured for up to 21 days and were monitored at timepoints 0, 12, 24, and 48 h, and on days 7, 14, and 21. This schedule was chosen due to the likelihood of extremely rapid rates of cell division during the first 24–48 h [15]. Medium was exchanged at 12, 24, and 48 h, and every 48 h thereafter. Three replicates were prepared for each cell density at each timepoint and sacrificed for counts.

### 4.5. Culture Methods

#### 4.5.1. 2-D Culture Controls

The 2-D cultures of *G. neptuni* cells were incubated in M1 medium to create low density (5 × 10^6^ cells/mL) and high density (1 × 10^7^ cells/mL) controls. In total, 30 µL of each inoculation cell density were added in triplicate to a 384-well microplate (Corning, Corning, NY, USA). The plates were then sealed and incubated at room temperature in the dark for 21 days. Monitoring occurred as described above. Cells were counted by pipetting the contents of each sacrificed well to resuspend the cells and then the cells were counted using an automated cell counter. As total medium replacement resulted in a loss of sponge cells, half medium exchanges were made at 12, 24, and 48 h, and every 48 h thereafter.

#### 4.5.2. 3-D Cultures: FibraCel^®^ Disks

FibraCel^®^ disks (Eppendorf, Enfield, CT, USA) are 0.5 cm flat, circular, support structures used in cell culture bioreactors. They are made of a polyester mesh with an attached polypropylene grid support. For the purposes of this experiment, the polypropylene grid was removed, as it renders the disks buoyant, and a knotted piece of polyester thread was strung through the center of the disk to provide an easy grip for manipulation of the disks. The disks were UV sterilized and placed at the bottom of corresponding wells of a 96-well plate. Cells were resuspended in M1 medium at the desired densities (1 × 10^7^, 5 × 10^6^ cells/mL), and 200 µL of each cell suspension were added to each well to allow cells to settle onto and attach to the FibraCel^®^ disks. After 24 h, the disks were transferred from the 96-well plate to a 24-well plate (BD, Franklin Lakes, NJ, USA), to provide a higher volume of nutrient media for the cells. The plates were sealed and incubated in the dark for 21 days. Medium exchanges were performed by transferring the disks to new wells containing fresh medium.

Cells were removed from the FibraCel^®^ disks for cell counts by incubating in 1 mL of calcium- and magnesium-free seawater (CMF) on an orbital shaker at 300 rpm for two hours, then agitated further by vigorous pipetting before automated counts were taken as described above. The disks were photographed after cell removal efforts to document the remaining cells still attached to the FibraCel^®^ disks at each time-point. The photographs were measured using Microsoft Digital Colorimeter software to determine the average color of the disks. This coloration was then used as a proxy to determine the extent to which cells were retained within the FibraCel^®^ disk.

#### 4.5.3. 3-D Cultures: Thin Hydrogel Layers

##### Porcine Gelatin Thin Hydrogel Layers

A gelatin solution was created as described above. The sponge cell suspension in gelatin was then pipetted (35 µL) into 96-well plates in triplicate to form a thin layer across the bottom of the well. The cultures were incubated at 4 °C for 30 min to allow the gelatin to cool and solidify. Following this incubation period, 100 µL of a 0.03% mTGase crosslinking solution were added to each well. The addition of mTGase forms intramolecular covalent bonds between polymers, which increases the stability of the gel layer. The plates were incubated at room temperature (~22 °C) for 30 min, after which the gel layers were washed with ASW. In total, 200 µL of medium M1 were added to each well and the plates were sealed (Thermo Fisher Scientific, Waltham, MA, USA) to prevent evaporation while maintaining gas exchange.

##### ULTA Thin Hydrogel Layers

Two ULTA-sponge cell suspensions were prepared to form final cell densities of 5 × 10^6^ cells/mL (low cell density) and 1 × 10^7^ cells/mL (high cell density) as described above. A total of 70 µL of each suspension were added to triplicate wells on 8 96-well plates. The ULTA was crosslinked as previously described, washed in ASW, and 200 µL M1 medium were added. The plates were sealed and incubated at room temperature in the dark for 21 days.

Cells were retrieved from the ULTA thin hydrogel layers for cell counts by removing the medium, adding 50 µL ASW to each well, and heating the plate to 70 °C for 30 min to melt the ULTA hydrogel. Then, 1 µL of 0.5% agarase (Thermo Fisher Scientific, Waltham, MA, USA) was added to each well and incubated at 70 °C for an additional 30 min to digest the ULTA. The resulting solution was resuspended by pipetting, and 10 µL of each culture were taken for automated cell counts.

#### 4.5.4. 3-D Cultures: ULTA GMDs

An ULTA suspension containing *G. neptuni* cells was prepared as described above and transferred to a 35 °C water bath consisting of a 40 × 80 mm petri dish (Thermo Fisher Scientific, Waltham, MA, USA) filled with water on a second hot plate to maintain a consistent temperature throughout droplet formation. Two ULTA-sponge cell suspensions were prepared to form final cell densities of 5 × 10^6^ cells/mL (low density) and 1 × 10^7^ cells/mL (high density). Droplets were prepared by pipetting 10 µL aliquots of the ULTA-sponge cell suspensions into chilled mineral oil. Pipette tips were exchanged between dispensing each droplet because the ULTA quickly solidified in the tip. The droplets were rinsed three times each by transferring them into a 50 mL centrifuge tube filled with 15 mL of chilled ASW. A new transfer pipette was used for each wash. Finally, the droplets were transferred into 96-well plates in groups of 5 GMDs per well, 3 wells (replicates) per plate. The GMDs were then suspended in 200 µL of M1 medium and the plates were sealed. Cultures were incubated in the dark at room temperature for 21 days. Cells were retrieved from the ULTA GMDs for counts by digesting the gel as described for the ULTA gel layers and conducting automated cell counts.

### 4.6. Data Analysis

Data for each inoculation density for each of the 3-D methods were compared to one another and to the 2-D control at maximum and final cell density using a two-tailed Student’s T-test with Bonferroni corrections for multiple pairwise comparisons; for all 3-D treatments, α = 0.017, and for the 2-D controls, α = 0.05.

## 5. Conclusions

Cells from the marine sponge *Geodia neptuni* were successfully cultured using three 3-D culture methods: FibraCel^®^ disks, ULTA thin hydrogel layers, and ULTA GMDs. These cultures performed comparably to 2-D control treatments, and there are merits to each culture type that recommend them for various applications. No cell differentiation was observed in any culture treatment, and further research is required to induce differentiation and sponge architecture formation.

The cause of the pigmentation observed when sponge cells are cultured in M1 medium needs to be addressed, and if possible, mitigated to prevent its interference in light-based analyses. This would broaden our ability to collect data on marine sponge cells and expand the types of studies that can be conducted using medium M1 and its derivatives, which are to date the only nutrient media capable of inducing cell division in marine sponge cells.

Continued research using the 3-D methods detailed here should focus on perfusion cultures to determine whether the cultures are nutrient limited. Finally, further research on scaling up these methods is recommended to increase their usefulness for application to production of sponge-derived chemicals with human health applications.

## Figures and Tables

**Table 1:** Comparison of 2-D treatments. There was no significant difference between treatments (α = 0.05).

	*p*-Values	
	Final Density	Maximum Density
2-D low density to 2-D high density	0.113	0.624

**Table 2:** Comparison between FibraCel^®^ treatments and with 2-D controls. The difference between low density FibraCel^®^ treatments and low density 2-D controls was statistically significant * at both maximum and final concentrations. (α = 0.017).

	*p*-Values	
	Final Concentration	Maximum Concentration
FibraCel^®^ low density to 2-D low density	0.011 *	0.005 *
FibraCel^®^ high density to 2-D high density	0.055	0.059
FibraCel^®^ high density to FibraCel^®^ low density	0.039	0.235

**Table 3:** Comparison between ULTA thin layer treatments and with 2-D controls. The differences between the high and low density ULTA treatments were statistically significant * at both the maximum and final cell concentration. (α = 0.017).

	*p*-Values	
	Final Concentration	Maximum Concentration
ULTA thin layer low density to 2-D low density	0.047	0.066
ULTA thin layer high density to 2-D high density	0.977	0.977
ULTA thin layer high density to ULTA thin layer low density	0.004 *	0.004 *

**Table 4:** Comparison between GMD treatments and with 2-D controls. No significant differences were found among any of the pairings (α = 0.017).

	*p*-Values	
	Final Concentration	Maximum Concentration
GMD low density to 2-D low density	0.036	0.582
GMD high density to 2-D high density	0.254	0.919
GMD low density to GMD high density	0.344	0.118

**Table 5:** Advantages and disadvantages of 3-D culture methods for culture of *Geodia neptuni* cells in M1 medium and recommended applications for production of biomass and/or marine natural products (MNPs). $ = low cost. $$ = moderate cost.

Culture Method	Advantages	Disadvantages	Effort	Expense	Recommended Applications
FibraCel^®^ disks	Hardy matrix retains cells	Unable to count cells	Low	$	Cell culture scale-up, aquaculture scale-up
ULTA thin hydrogel layers	Easy to visualize cells/aggregates	Delicate matrix	Mid	$$	Cell characterization and differentiation, aquaculture scale-up
ULTA GMDs	Rapid diffusion of nutrients, gases and products	Delicate matrix	High	$$	Cell metabolism studies, cell culture scale-up

## Data Availability

The data presented in this study are available on request from the corresponding author.

## Appendix A. Analysis of Protein Concentration in Sponge Cells

As it was difficult to remove all the cells from the FibraCel Disks© disks, analysis of total protein content was attempted as a proxy for cell counts. Protein was extracted using Radio Immunoprecipitation (RIPA) Lysis and Extraction Buffer (Thermo Fisher Scientific, Waltham, MA, USA), which contained Halt^TM^ Protease and Phosphatase Inhibitor Single-Use Cocktail (Thermo Fisher Scientific, Waltham, MA, USA). Sponge samples and bovine serum albumin (BSA) standards were pipetted in triplicate into a 96-well plate and combined with a BCA working solution by mixing on an orbital shaker for 30 s. The plate was then incubated at 37 °C for 30 min. A Synergy H1 plate reader (BioTek) was used to take measurements at 562 nm absorbance. The measurements for each BSA standard were blank-corrected and averaged before plotting them against their concentration (µg/mL) to create a standard curve, which was then used to determine the protein concentration for each sample.

Protein was successfully extracted from cryopreserved *G. neptuni* cells (3.55 × 10^6^ cells/mL) immediately upon removal from −80 °C storage. Total protein extracted was 133.37–138.11 µg/mL (BioTek [Winooski, VT, USA] Synergy H1 plate reader, 562 nm absorbance). Although several attempts were made to measure protein extracted from cells that had been 2-D cultured in medium M1 for 24 h, all measurements obtained from the plate reader were <0.00, indicating that the instrument was unable to obtain quantifiable protein measurements for these samples. Therefore, total protein content analysis could not be used to quantify an increase in cell number in the FibraCel^®^ disks.
